# An Essay on the Common Cause and Prevention of Hepatitis, or Disorder of the Liver; and of Bilious Complaints in General, as Well in India as in Europe

**Published:** 1817-02

**Authors:** 


*An Essay on the Common cause and Prevention of Hepatitis, or Disorder of the Liver; and of Bilious Complaints in general, as well in India as in Europe.* By CHARLES GRIFFITH, M. D. Senior Surgeon to the Forces, &c. Octavo pp. 202. London, 1816.

Dr. Griffith's object, we believe, is not self interest, as is evidently the case in Mr. Faithorne's trumpeted book "on Liver Complaints." But it is a very meagre performance, and, at all events, is not designed for the medical reader. We think Dr. G. might have produced a better work at his time of life (upwards of 60); and what is more, we think that he has formed a most erroneous notion of his grand panacea (exercise) in hot climates. From no limited experience, we know that exercise is the cause, in a very great proportion of cases, of liver complaints; and the immunity from this complaint which females so generally enjoy, arises, we are convinced, from their more sedentary habits, in conjunction with temperance.

An appendix, addressed to the profession, contains a long and confused declamation against the calomel now in use, and recommeuding the old submuriates in its stead. We do not think it necessary to fill up any space in our Journal with observations on this appendix; it is on a par with the popular part of the work. We shall quote a single passage, which is the last in the work, and probably the best.

"In this obstruction, (the ague cake or enlarged spleen so common after protracted intermittents and remittents) I must confess, mercury, contrary to the above rule, is so insignificant, that I have sometimes on that account been almost inclined to doubt the *glandular* construction of this viscus, all other glands evincing its deobstruent power.

"Being first disappointed in two cases which occurred to me of this description at Calcutta, in each of which I had twice excited the salivary glands to no purpose, the enlargement still increasing, I was urged by the dread of suppuration to make trial of the *succus conii inspissatus*, which I persisted in, having luckily preserved some in my own little stores, until I had repeatedly affected the head, the tumour in both cases gradually lessening, until at length it was wholly done away: had the above been insufficient to establish the good effects of the *conium*, the expedition to Walcheren (during and subsequent to which this disease prevailed but too fatally) afforded me ample proof of its efficacy." p. 202.

